# Consultation on UTUC II Stockholm 2022: diagnostics, prognostication, and follow-up—where are we today?

**DOI:** 10.1007/s00345-023-04530-9

**Published:** 2023-08-04

**Authors:** Filip Sydén, Joyce Baard, Matthew Bultitude, Francis Xavier Keeley, Morgan Rouprêt, Kay Thomas, Tómas Andri Axelsson, Georg Jaremko, Helene Jung, Camilla Malm, Silvia Proietti, Palle Jørn Sloth Osther, Marianne Brehmer

**Affiliations:** 1 Department of Urology, Stockholm South General Hospital, Stockholm, Sweden; 2 Department of Urology, Amsterdam UMC, University of Amsterdam, Amsterdam, The Netherlands; 3 Cancer Center Amsterdam, Amsterdam UMC, Amsterdam, The Netherlands; 4 Guy’s and St. Thomas’ NHS Foundation Trust, Urology Centre/Stone Unit, Guy’s and St. Thomas’ Hospital, London, UK; 5 Bristol Urological Institute, North Bristol NHS Trust, Bristol, UK; 6 Sorbonne University, Pitié Salpétrière Hospital, Assistance Publique Hôpitaux de Paris, Paris, France; 7 Department of Surgery and Urology, Danderyd Hospital, Stockholm, Sweden; 8 Department of Oncology and Pathology, Karolinska Institute, Stockholm, Sweden; 9 Department of Urology, Lillebaelt Hospital, University Hospital of Southern Denmark, Vejle, Denmark; 10 Urology Department, San Raffaele Hospital, Milan, Italy; 11 Department of Clinical Sciences Karolinska Institute, Stockholm, Sweden

**Keywords:** Upper tract urothelial carcinoma, UTUC, Diagnostics, Prognostication, Follow-up, Computed tomography urography, Ureterorenoscopy, Cytology, Biopsy

## Abstract

**Purpose:**

To summarise the current knowledge regarding diagnostics, prognostication and follow-up in upper tract urothelial carcinoma (UTUC).

**Methods:**

A scoping review combined with expert opinion was applied to provide an overview of the current research field. Based on the published literature and the experts’ own experience and opinions, consensus was reached through presentations and discussions at the meeting Consultation on UTUC II in Stockholm 2022.

**Results:**

The strongest prognostic factors in UTUC are tumour grade and stage. They are correlated, and grade is used for indirect staging. The diagnostic examinations should include multiphase computed tomography urography (CTU) with corticomedullary phase, and urethrocystoscopy with cytology. If there is no clear diagnosis for clinical decision-making, ureterorenoscopy (URS) with focal cytology and biopsies should be performed. Both WHO classification systems (1973/1999 and 2004/2016) should be used. Novel biomarker tests are not yet widespread nor recommended for the detection of UTUC. Long-term, regular follow-up, including URS in patients who have had organ-sparing treatment, is important to check for tumour recurrences, intravesical recurrences, metastases and progression of the tumour.

**Conclusion:**

Proper diagnostics with correct grading of UTUC are necessary for appropriate treatment decisions. The diagnostics should include CTU with corticomedullary phase, urine or bladder cytology, URS with focal barbotage cytology, and biopsies when needed for proper diagnosis and risk stratification. Regular, long-term follow-ups are fundamental, due to the high rate of recurrence and risk of progression.

## Introduction

Urothelial carcinoma (UC) is the sixth most common cancer in developed countries. Upper tract urothelial carcinoma (UTUC) in the renal pelvis or the ureter is comparatively rare and represents 5–10% of UC with an estimated incidence of 2/100,000 in Western countries [1]. The female/male ratio of incidence is 1:2 [2]. At diagnosis, 1.6% of patients have synchronous bilateral UTUC; however, 2–6% later develop contralateral UTUC [3, 4]. Risk factors for the development of UTUC are not only environmental factors such as smoking and intake of aristolochic acid, but also hereditary factors where Lynch syndrome is the most common [5–7]. The EAU Guidelines classify UTUC into low-risk and high-risk UTUC according to certain criteria based on current knowledge. In the low-risk group, organ-sparing treatment is recommended, whereas radical nephroureterectomy (RNU) is the treatment of choice in organ-confined high-risk UTUC [8]. The AUA Guidelines have basically taken a somewhat different classification of UTUC where tumour grade is the key. UTUC is divided into two presurgical clinical risk categories based on the tumour grade: low-risk or high-risk UTUC. Both categories are sub-stratified into favourable or unfavourable based on additional variables (radiographic findings, unifocality or multifocality, involvement of lower urinary tract) and recommendations regarding ablative treatments and systemic therapy are proposed based on the risk classification [9].

Furthermore, there must be a clear aim of organ-sparing treatments: elective, relative, imperative, or palliative. For palliative organ-sparing treatment, indications may be to achieve local disease control, or to give guidance regarding what systemic therapy may be indicated whilst the patient still has preserved renal function.

The 5-year disease-specific survival (DSS) for patients with UTUC is relatively low compared to patients with UC in the bladder as approximately 60% of the patients have invasive cancer at the time of diagnosis and approximately 9% have metastases [6, 10]. Since UTUC is rare and prospective randomised controlled studies are lacking, the recommendations in the EAU Guidelines have limitations.

The objective of Consultation on UTUC II Stockholm 2022 was to assemble expert clinicians and scientists within the field to discuss present guidelines, share current knowledge on diagnostics, prognostication, and follow-up regimens. Based on the published literature and the experts’ own experience and opinions, consensus was reached through presentations and discussions at the meeting.

## Diagnostic workup

Accurate diagnostic workup is important to optimise risk stratification and minimise suboptimal or over-treatment of patients with UTUC. In the diagnostic workup, multiphase computed tomography urography (CTU) including the corticomedullary phase is the recommended imaging modality for diagnostics and staging [11]. Urethrocystoscopy with cytology is recommended to exclude concurrent UC in the bladder, which is present in 17% of cases at diagnosis [12]. Malignant cells in cytology (urine or at urethrocystoscopy) despite no findings in the bladder and urethra may indicate UTUC. If imaging with CTU and cytology from passed urine or the bladder are not sufficient for clear diagnosis and risk-group categorisation, the EAU Guidelines as well as the AUA Guidelines recommend performing a diagnostic ureterorenoscopy (URS), where it is possible to take biopsies and collect focal barbotage samples for cytology analysis. However, diagnostics with URS should not delay the time of performing RNU, which is recommended to be within a maximum of 12 weeks of diagnosis, which of course is depending on how far the cancer disease has progressed at the time of diagnosis [13, 14]. In patients with non-ambiguous high-grade (HG) malignant cells in voided urine cytology, and no contraindications for radical surgery, the patients should directly be treated with RNU, without prior URS with biopsy.

Consensus reached at the Consultation on UTUC II Stockholm 2022: diagnostic URS should be performed if imaging with CTU and cytology are not sufficient for clear diagnosis or risk classification.

## Radiological examinations

**Fig. 1:**
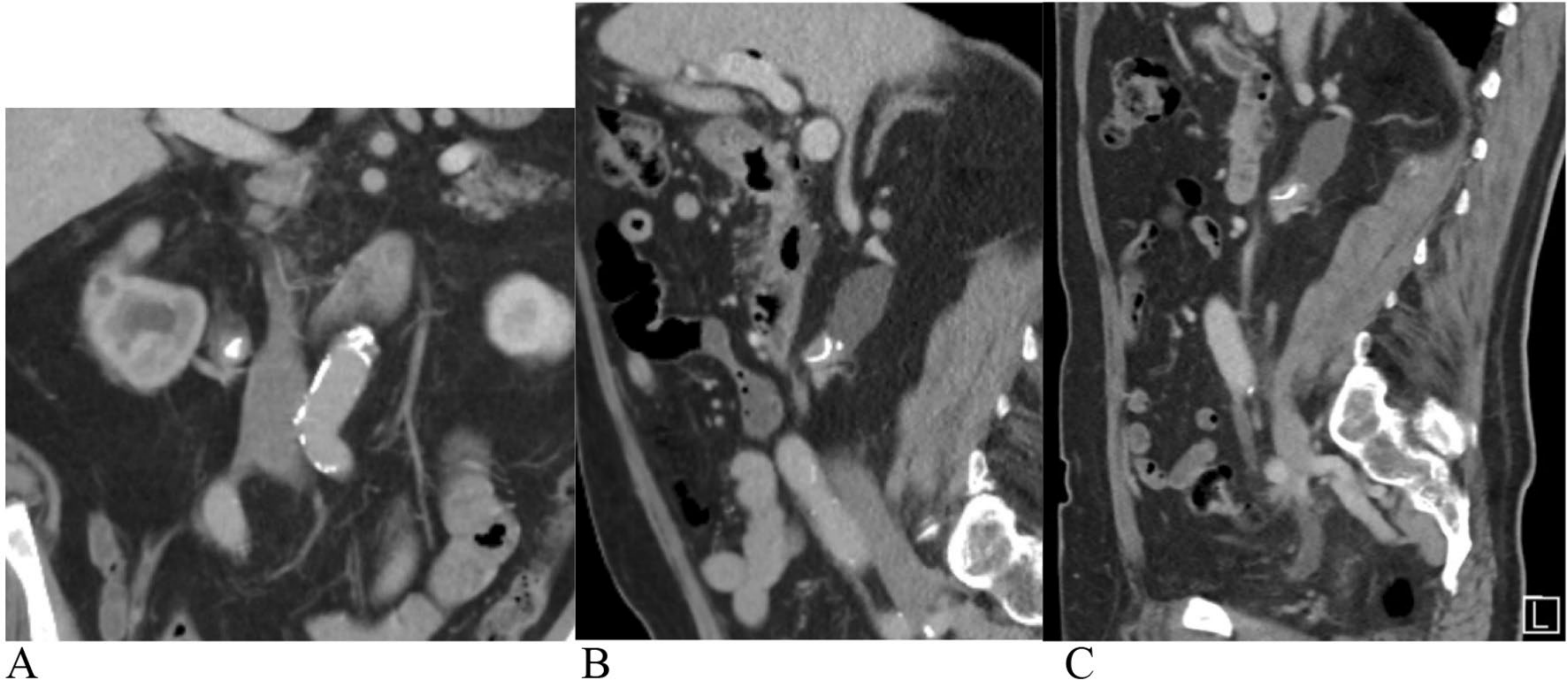
A 79-year-old man with right sided flank pain. No other symptoms. Previous smoker. A split bolus CT scan was performed. A calcification was found in the pelvoureteric junction (PUJ) and the patient was diagnosed as having a renal stone. Split bolus CT scan has a native phase and then contrast is given as two doses, so that there are simultaneous venous and excretion phases, which is not optimal to find urothelial tumours. After 2 months, the patient was referred to the urology department and a multiphase CTU was performed. **a** Split bolus coronar view. A calcification can be seen in the PUJ. **b** Split bolus sagittal view. A calcification can be seen; however, it is formed like an eggshell. **c** Multiphase CTU, sagittal view. A contrast loading tissue mass can be seen around the calcification. URS revealed a tumour with calcification on the surface

It can be challenging to assess invasiveness of UTUC with CTU, unless the tumour is advanced. However, for staging, CTU is informative regarding lymph node involvement and distant metastasis. In the upper urinary tract, CTU has a sensitivity of 89% for detecting UTUC; however, with smaller lesions, the sensitivity is lower, decreasing to 40% for lesions sized less than 3 mm [15]. When performing CTU, it is important to use a multiphase CTU (three or four phases), since the optimal phases for discovering tumour lesions are corticomedullary and urothelial phase, as shown in Fig. 1 [16, 17]. The shortcomings of CTU are that it does not give a pathological diagnosis, involves ionising radiation and requires intravenous contrast with the potential side effects, for example, allergic reaction or acute kidney injury. In cases where CTU is contraindicated, magnetic resonance (MR) urography is indicated despite the lower sensitivity of 75% [18, 19]. Examination techniques to enhance sensitivity for MR urography have been tested, including diffusion-weighted sequences to calculate the apparent diffusion coefficient (ADC) parameter [20]. The PI-RADS protocol for prostate cancer has been transformed to the VI-RADS protocol in detecting UC in the bladder but has not yet been validated for UTUC. The use of positron emission tomography with computed tomography (PET-CT) for lymph node staging is still under evaluation, but looks promising for the future diagnostics of UTUC, since a sensitivity of 82–95% and specificity of 84–91% for lymph node staging have been reported in a systematic review [21, 22].

Consensus reached at the Consultation on UTUC II Stockholm 2022: the diagnostic examination should include multiphase CTU including corticomedullary phase if no contraindications are present.

## Ureterorenoscopy, URS

Diagnostic URS provides information regarding tumour characteristics such as localisation, size, architecture and focality and enables collection of samples for cytology and histopathology [23, 24]. In a retrospective study by Golan et al., 24% of patients with suspicious malignant findings on CTU had benign findings on diagnostic URS and, hence, would have risked being overtreated with RNU had URS not been performed [25]. In another study, the rate of RNU declined from 89 to 69% when diagnostic URS was performed, and the incorrect diagnosis rate after RNU decreased from 15.5 to 2.1% [26]. In a retrospective review from Scotland et al., the complication rate was 7.1% when URS was performed, including urinary tract infections, sepsis, and ureteral stricture [27]. Another review of 38 studies with more than 1100 patients, and reported complications from URS in UTUC, showed ureteral stricture as the major complication, reaching as high as 27% and correlating with the number of procedures patients had undergone [28]. When adjuvant treatment with BCG or mitomycin C was added, haemorrhage, infection and fever were observed to be the most frequent complications. Severe and lethal complications were rare [28].

The development of flexible ureteroscopes with digital technology has ameliorated both diagnostic and therapeutic URS. As the ureter is narrow and long, instruments must be sized in a way to fully access the whole urinary tract system, but still be able to capture digital images and harbour enhancement technologies [29]. Techniques to improve diagnostics and enhance tumour visualisation in URS are narrow-band imaging (NBI), Storz professional imaging enhancement system (SPIES/Image1-S) and photodynamic diagnosis (PDD) [30]. Data from the CROES-UTUC registry, however, showed that the impact of these techniques is unclear [31]. Other methods to obtain intraoperative real-time information on tumour grade and stage are optical coherence tomography (OCT), confocal laser endomicroscopy (CLE) and endoluminal ultrasound (ELUS) [32–34]. They are not used routinely and have not provided as much of a practise transformation as anticipated.

Consensus reached at the Consultation on UTUC II Stockholm 2022: diagnostic URS should be performed when needed for proper diagnosis and risk stratification.

## Biopsy for histopathology

The objective of taking biopsies is to unveil tumour grade, which may be used for indirect tumour staging as well, and thus is very important for treatment decision [35]. Local stage usually cannot be defined from biopsies. However, there is a strong correlation between grade and stage, enabling indirect staging [3, 8, 36]. The correlation is stronger using the WHO 1973/1999 than the WHO 2004/2016 classification system [3].

**Fig. 2:**
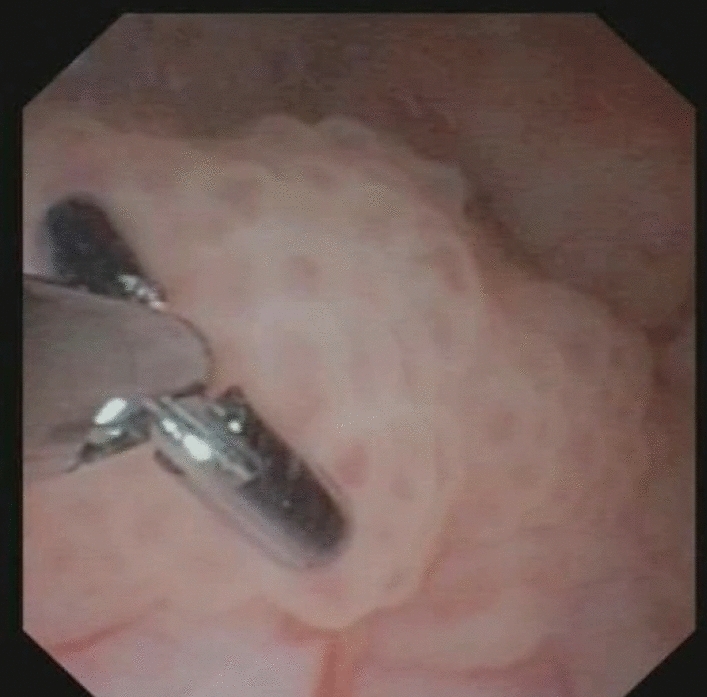
A tumour biopsy from the renal pelvis is taken using a flexible ureteroscope and biopsy forceps

Before performing organ-sparing treatment, it is fundamental to rule out high-grade lesions. Retrieving URS biopsies may be challenging; the narrow working channel in the most frequently used flexible ureteroscopes is 3.6 Fr, and the forceps must be thinner, as shown in Fig. 2 [37]. Putting the tiny specimen in Bouin's solution may be a useful alternative to formalin, as it provides excellent nuclear detail to assist in accurate grading [38]. Histopathology from biopsies can detect tumour grade in 69–90% of cases, although there is a risk of under grading due to tumour heterogeneity [39–41]. Therefore, selective in situ cytology is an important complement to biopsies [42]. Adding the findings from CTU to the information from URS with cytology grade and biopsies may assist in deciding what treatment is to be chosen for a specific patient [43].

Consensus reached at the Consultation on UTUC II Stockholm 2022: endoscopically taken biopsies are of high importance to unveil tumour grade and are highly valuable for proper risk classification.

## Cytology

Cytology for the detection of malignant cells in UTUC can be performed using voided urine, selective ureteral catheterisation, or intraoperative collection of saline barbotage fluid. Due to the heterogeneity of the tumour, grading can be improved with cytology, which potentially represents a larger part of the tumour. For voided urine, the sensitivity is rather low—43% to detect grade 2 malignant cells and 80% for grade 3 (WHO 1973/1999) [44]. Cytology analysis of urine from selective ureteral catheterisation has a sensitivity of 65% for grade 2 cells and 96% for grade 3 cells [45]. The usefulness of selective ureteral catheterisation was investigated in a meta-analysis, where the pooled sensitivity of selective cytology stratified by tumour grade was 45.6% for low-grade tumours, and 69.9% for high-grade tumours [46]. However, by performing in situ saline barbotage cytology, a 91% sensitivity in identifying UTUC of all grades has been reported [47]. In another study from Zhang et al., they found washings from the upper urinary tract outperformed voided urine in the detection of HG UTUC [48]. Thus, the way samples for cytology analysis are collected seems very important for detection of UTUC. The classification of urinary cytology was standardised in the Paris System, with priority of the identification of HG malignant cells and decreasing the uncertainty of non-HG findings. In the Paris System for reporting cytology, findings are divided into seven categories [49]. It can be reported in combination with the WHO 1973/1999 classification.

Consensus reached at the Consultation on UTUC II Stockholm 2022: due to the heterogeneity of the tumour, grading can be improved with cytology. The Paris system should be used to report cytological findings.

## Alternative tests for UTUC on the market

There are several biomarker tests on the market for the detection of UC using analysis of proteins, DNA and mRNA in urine or blood. Series have been reported in UTUC for use of several molecular tests including FISH, BTA, NMP22, uCyt, Bladder EpiCheck and Xpert Bladder Cancer, although all these series are small with variable sensitivity and specificity [50]. They might provide extra information as complement to histopathology at diagnosis and follow-up, and, thus, potentially may reduce the need of invasive procedures for diagnosis. However, they are neither widespread nor recommended in the EAU Guidelines yet, since they are developed for UC of the bladder, and more validation for UTUC is needed.

Consensus reached at the Consultation on UTUC II Stockholm 2022: biomarker tests might provide extra information as a complement to histopathology at diagnosis and follow-up, but still need further evaluation.

## Prognostication

Prognostic factors to aid treatment decision are of utmost importance. Tumour stage and grade are prognostic factors with strong evidence in UTUC, whereas other criteria in the EAU Guidelines are supported by less strong evidence. Thus, proper diagnostics with correct grading and staging are important for appropriate treatment decisions [36].

As mentioned before, there is a strong correlation between grade and stage. UCs are currently classified according to two classification systems: WHO 1973/1999, by which tumour grade is categorised as grades 1–3, and WHO 2004/2016, by which UC is described as either low grade or high grade and arrived when WHO adopted the International Society of Urological Pathology (ISUP) criteria, with new stratification of the different categories [51, 52]. The WHO 1973/1999 classification has been reported to be superior to the WHO 2004/2016 in prognostic value for cancer-specific survival in UTUC [53]. This is in line with a multicentre study on UC in the bladder resulting in a recommendation to use a combination of the classification systems, creating a four-tiered system of grade 1, grade 2 low-grade, grade 2 high-grade, and grade 3 [54].

In the EAU Guidelines, several known prognostic factors of survival are stated, such as lower cancer-specific survival with old age, tobacco consumption, comorbidity and performance status [55, 56]. To try to improve the accurate risk stratification prior to treatment, several preoperative risk nomograms have been tested, but they are not yet in widespread use [57]. The EAU risk stratification has changed over time, and new studies propose further improvement. For example, less stress on multifocality and size, but instead adding age, stage from biopsies and architecture of tumour [36, 58]. More focus on grade assessed by URS specimens, and stage assessed by CTU or MR urography is also suggested [59]. According to the EAU Guidelines, the recommendation to use prognostic factors to risk-stratify patients for therapeutic guidance has a weak strength rating [8]. The AUA Guidelines state that risk stratification into low-risk or high-risk UTUC should be performed based on grade from biopsies. Both categories are then sub-stratified into favourable or unfavourable, based on findings from URS such as architecture and focality, and results from cytology. Important findings from radiology include signs of invasion, obstruction, and lymph node staging. Also, lower urinary tract involvement is of importance, both concomitant and former. From these findings, recommendations regarding ablative treatments and systemic therapy are proposed [9]. Tumour size is considered having less clinical importance in the AUA Guidelines, since data regarding size have been derived from histopathology after resection and not from preoperative radiology or URS [57].

Consensus reached at the Consultation on UTUC II Stockholm 2022: proper diagnostics, especially with correct grading, is necessary for appropriate treatment decisions.

## Follow-up after UTUC treatment

The aim of follow-up is to check for upper tract tumour recurrences, intravesical recurrences (IVR), metastases and progression of tumour stage and grade. If organ-sparing treatment has been performed, follow-up including long-term surveillance with repeated CTU, urethrocystoscopy and URS is essential [60]. The EAU Guidelines regarding follow-up are brief due to the fact that there is little evidence or consensus, and thus follow-up regimes need to be individualised to the patient. There is high recurrence rate, and several patients develop IVR [61]. It has been proposed as a bare minimum to perform a second look URS 6–8 weeks after initial and complete tumour ablation, since it has shown increased recurrence-free survival (RFS) and a conversion rate, from organ-sparing treatment to RNU, of 19.5% after repeated URS [62]. However, the EAU Guidelines do recognise that “repeated endoscopic procedures are necessary”, after organ-sparing treatment. Reports of structured long-term follow-up after organ-sparing treatment of UTUC are scarce. However, in a retrospective study from Scotland et al. [27], patients were followed for a period of 23 years, and the mean follow-up time was 5.5 years. The follow-up protocol consisted of URS and urethrocystoscopy every 3 months until clearance of recurrent tumour, then every 6 months until 5 years, and thereafter once a year. Tumour recurrence resets the follow-up protocol. They reported a 5-year cancer-specific survival of 92.6%, and an RFS of 30%. In the study, up to 25% of the patients had tumour progression at 5 years follow-up. The kidney preservation rate in the study was 71.4%, which highlights the need for strict follow-up. Recurrence or progression occurred in some cases after more than 5 years, underlining the importance of a long-term follow-up. Compared to patients who underwent RNU, patients treated with organ-sparing surgery have been reported to have a higher incidence of IVR of 68% [63]. However, IVR does not seem to have an impact on cancer-specific survival [64].

Consensus reached at the Consultation on UTUC II Stockholm 2022: regular and long-term follow-up, including URS if organ-sparing treatment has been carried out, is crucial for handling recurrences, IVRs, metastases and tumour progression.

## Conclusion

Proper diagnostics with correct grading are necessary for accurate risk stratification, thereby enabling treatment decisions. The diagnostic workup should include multiphase CTU with corticomedullary phase and urine cytology or cytology taken at cystoscopy. URS with focal cytology, preferably taken as a barbotage, and biopsies may add valuable diagnostic information, which should not be underestimated. Both WHO classification systems (1973/1999 and 2004/2016) should be used due to their added prognostic value. Biomarker tests on the market are not yet recommended in the EAU Guidelines, since they are developed for UC of the bladder, and more validation for UTUC is needed. Regular and long-term follow-up, including URS if organ-sparing treatment has been carried out, is crucial for handling recurrences, IVRs, metastases and tumour progression. During the Consultation on UTUC II in Stockholm, discussions were held regarding the recommendations in the EAU Guidelines for the follow-up of patients after organ-sparing treatments. They were considered to represent the bare minimum, and it was suggested they should be updated with more stringent recommendations regarding length and frequency of the follow-up, bearing in mind the high rate of recurrence and risk of progression. The AUA Guidelines state from expert opinion that follow-up should be performed longer than 5 years in the absence of recurrence, based on shared decision-making between the patient and clinician. In case of a high-risk tumour, a follow-up exceeding 5 years is encouraged [9, 60].
